# Interaction between Functional Connectivity and Neural Excitability in Autism: A Novel Framework for Computational Modeling and Application to Biological Data

**DOI:** 10.5334/cpsy.93

**Published:** 2023-01-20

**Authors:** Yuta Takahashi, Shingo Murata, Masao Ueki, Hiroaki Tomita, Yuichi Yamashita

**Affiliations:** 1 Department of Psychiatry, Tohoku University Hospital, Japan; 2 Department of Psychiatry, Graduate School of Medicine, Tohoku University, Japan; 3 Department of Information Medicine, National Center of Neurology and Psychiatry, Japan; 4 Department of Electronics and Electrical Engineering, Faculty of Science and Technology, Keio University, Japan; 5 School of Information and Data Sciences, Nagasaki University, Japan

**Keywords:** Computational psychiatry, recurrent neural network, autism spectrum disorder, predictive coding, functional magnetic resonance imaging, functional connectivity

## Abstract

Functional connectivity (FC) and neural excitability may interact to affect symptoms of autism spectrum disorder (ASD). We tested this hypothesis with neural network simulations, and applied it with functional magnetic resonance imaging (fMRI). A hierarchical recurrent neural network embodying predictive processing theory was subjected to a facial emotion recognition task. Neural network simulations examined the effects of FC and neural excitability on changes in neural representations by developmental learning, and eventually on ASD-like performance. Next, by mapping each neural network condition to subject subgroups on the basis of fMRI parameters, the association between ASD-like performance in the simulation and ASD diagnosis in the corresponding subject subgroup was examined. In the neural network simulation, the more homogeneous the neural excitability of the lower-level network, the more ASD-like the performance (reduced generalization and emotion recognition capability). In addition, in homogeneous networks, the higher the FC, the more ASD-like performance, while in heterogeneous networks, the higher the FC, the less ASD-like performance, demonstrating that FC and neural excitability interact. As an underlying mechanism, neural excitability determines the generalization capability of top-down prediction, and FC determines whether the model’s information processing will be top-down prediction-dependent or bottom-up sensory-input dependent. In fMRI datasets, ASD was actually more prevalent in subject subgroups corresponding to the network condition showing ASD-like performance. The current study suggests an interaction between FC and neural excitability, and presents a novel framework for computational modeling and biological application of a developmental learning process underlying cognitive alterations in ASD.

## Introduction

There are accumulating reports of alterations in functional connectivity (FC) and neural excitability in autism spectrum disorder (ASD) (Dickinson et al., 2016; Hull et al., 2017; Itahashi et al., 2014; Vasa et al., 2016). However, studies that have examined these phenomena have also reported inconsistent results (Dickinson et al., 2016; Hull et al., 2017; Vasa et al., 2016). These inconsistencies may be due in part to the fact that FC and neural excitability interact in a complex way to affect ASD symptoms (Hegarty et al., 2018; Siegel-Ramsay et al., 2021; Vasa et al., 2016).

For such issues, computational psychiatry is expected to contribute by examining information processing alterations that cause psychiatric symptoms at the system level (Friston et al., 2014). In computational psychiatry, predictive processing (or predictive coding) theory is one of the most promising computational theories of perception and cognition (Friston et al., 2014). Predictive processing theory is linked to findings of altered neural activity in ASD, such as FC and neural excitability, by hypotheses and explanations presented using a neural network modeling approach (Idei et al., 2018; Idei et al., 2020; Idei et al., 2021; Takahashi et al., 2021). However, biological validation of these hypotheses is still challenging. This is because, while there are some challenges (Cross et al., 2021; Friston et al., 2003), there is no well-established methodology to validate simulation of a developmental learning process in which multiple parameters interact to cause ASD symptoms.

In the current study, we propose a novel framework for neural network modeling research that is followed by a biological application study. We first perform neural network simulation focusing on model parameters, which are assumed to be related to ASD, i.e., FC and neural excitability. Then, we investigate whether these parameters interact to cause ASD-like performance, i.e., failures in facial emotion recognition or generalization. Next, results of the neural network simulation were applied to actual functional magnetic resonance imaging (fMRI) datasets from ASD patients. By assuming that FC and neural excitability parameters of the neural network model correspond to functional connectivity and regional homogeneity (ReHo) (Zang et al., 2004) in fMRI, a neural network model with particular parameter conditions is assumed to represent a particular subject with corresponding brain characteristics of fMRI. We then examine whether subject subgroups corresponding to neural networks that exhibit ASD-like performance actually show ASD diagnoses/symptoms.

## Methods

### Overview of neural network simulation

**Figure 1:**
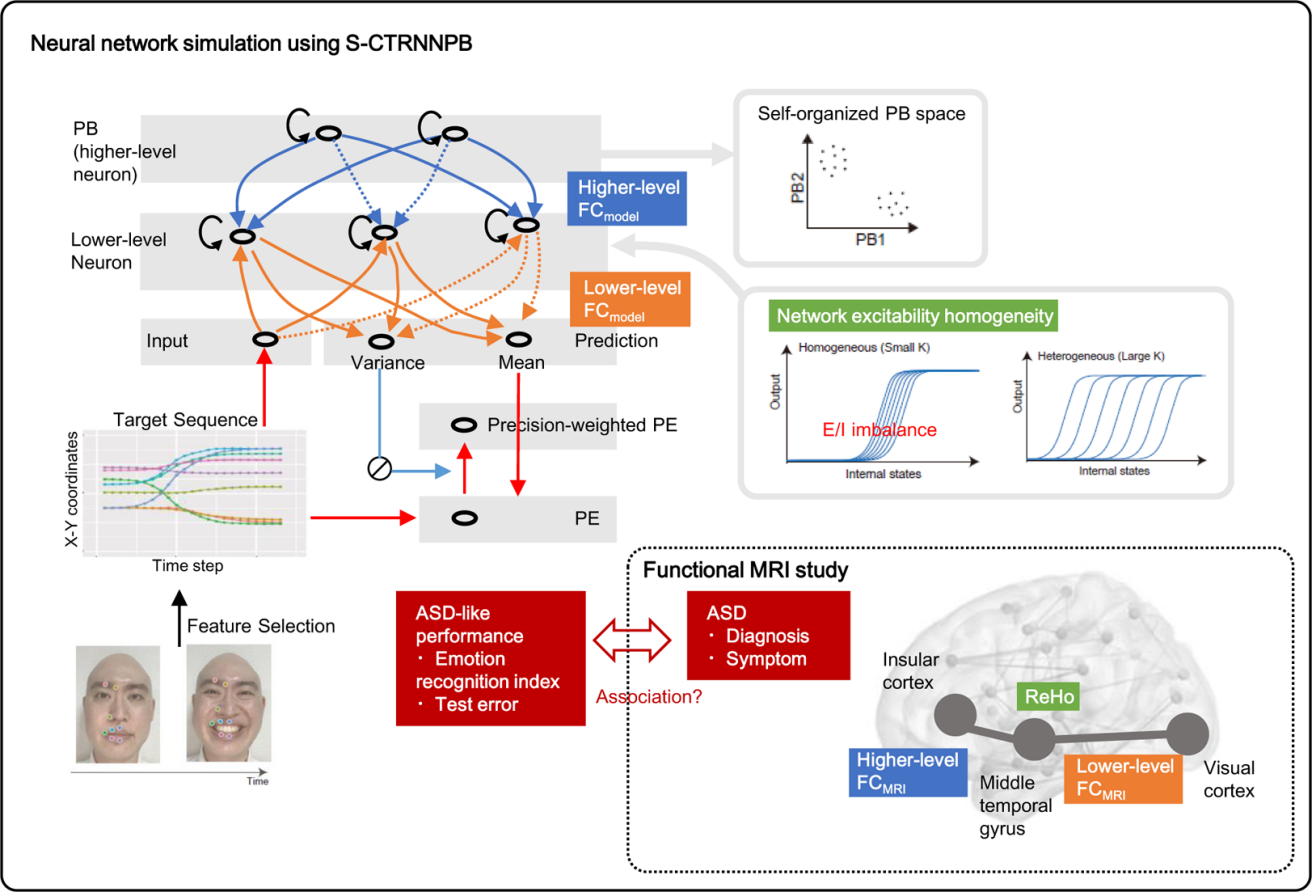
Framework of the current study and network architecture. The research framework consists of a simulation study and an fMRI study. First, we performed a simulation study using S-CTRNNPB, which models a biological brain based on predictive processing theory. Effects of S-CTRNNPB parameters (experimental conditions) on ASD-like performance were investigated. Second, we mapped the S-CTRNNPB parameter set to the fMRI parameter set. We then examined whether the relationship between the S-CTRNNPB parameters and ASD-related performance measures could also be found between fMRI parameters and ASD diagnosis/symptoms. Experimental conditions for the simulation study are FCmodel and Neural excitability homogeneity. FCmodel is the proportion of synaptic connections between neurons of different hierarchical level, set at 20–100%. In the figure, solid and dashed blue (orange) arrows represent the presence and absence of synaptic connections between neurons, respectively. Synaptic connections between neurons of the same hierarchy are unified in all experiments; there is no connection between PBs and full-connection between lower-level neurons. Neural excitability homogeneity is defined by the variance of the activity threshold of the lower-level neuron. See the Methods for details on experimental conditions. For the model architecture, the number of PBs and lower-level neurons are set to 2 and 30, respectively. Note that these neurons model the firing frequency of a population of neurons, not the activity of individual neurons in the biological brain. See the Supplementary Methods for details on parameter setting. *Abbreviations*. S-CTRNNPB, stochastic continuous time recurrent neural network with parametric bias; PB, parametric bias; PE, prediction error; ASD, autism spectrum disorder; MRI, magnetic resonance imaging; FC, functional connectivity; ReHo, regional homogeneity.

The framework of the study is described in Figure 1. The neural network model is trained to minimize the precision-weighted prediction error of the next step value in the target sequence obtained from facial expression videos, based on predictive processing theory. It is important to note that no emotion labels are given to this model. Whether this model is able to recognize emotions is evaluated by observing the “self-organization” of clusters for each emotion in the higher-level neuron, i.e., parametric bias (PB) space, which will be explained later. The experimental procedure for evaluating ASD-like performance consists of a training phase, which is analogous to the developmental learning process, and a test phase, which is analogous to the emotion recognition process from unknown facial expressions.

### Neural network model

The main component of the neural network model used in the simulation is the stochastic continuous time recurrent neural network with parametric bias (S-CTRNNPB), which is analogous to the biological brain, based on predictive processing theory (Murata et al., 2013; Murata et al., 2017). In the feed-forward prediction of S-CTRNNPB, the internal state of the *i*th neuron at time step *t* for the *s*th sequence is calculated as follows,

1 \[u_{t,i}^{\left(s \right)} = \left\{ {\begin{array}{*{20}{l}}
{u_{t - 1,i}^{\left(s \right)}}&{i \in {I_P}}\\
{\frac{1}{{{\tau _i}}}\left({\mathop \sum \limits_{j \in {I_I}} {w_{ij}}x_{t,j}^{\left(s \right)} + \mathop \sum \limits_{j \in {I_L}} {w_{ij}}l_{t - 1,j}^{\left(s \right)} + \mathop \sum \limits_{j \in {I_P}} {w_{ij}}p_{t,j}^{\left(s \right)} + {a_i}} \right) + \left({1 - \frac{1}{{{\tau _i}}}} \right)u_{t - 1,i}^{\left(s \right)}}&{i \in {I_L}}\\
{\mathop \sum \limits_{j \in {I_L}} {w_{ij}}l_{t,j}^{\left(s \right)} + {a_i}}&{i \in {I_M},{I_V}}
\end{array}} \right.\]

where *I_I_, I_P_, I_L_, I_M_*, and *I_V_* are index sets of the input, parametric bias (PB), lower-level, predicted mean, and variance neurons, respectively; *w_ij_* is the synaptic connection weight from the *j*th neuron to the *i*th neuron; \[x_{t,j}^{(s)}\] is the *j*th external input value at time step *t* of the *s*th sequence, \[l_{t,j}^{(s)}\] is the *j*th lower-level neuron, \[p_{t,j}^{(s)}\] is the *j*th PB activity; *τ_i_* is the time constant of the ith neuron; and *a_i_* is the activity threshold of the th neuron. As represented in the above equation, S-CTRNNPB is a hierarchical neural network model, and higher-level neuron is called PB. Equation (1) indicates that the internal state of PB neurons does not change with time.

Parameter optimization is performed by minimizing the negative log-likelihood, which assumes a Gaussian distribution for observations, as shown in the following equation,

2 \[L_{t,i}^{\left(s \right)} = \frac{{\ln \left({2\pi v_{t,i}^{\left(s \right)}} \right)}}{2} + \frac{{{{\left({\hat y_{t,i}^{\left(s \right)} - y_{t,i}^{\left(s \right)}} \right)}^2}}}{{2v_{t,i}^{\left(s \right)}}}\]

where \[v_{t,i}^{(s)}\] and \[y_{t,i}^{(s)}\] are the predicted mean and variance, respectively; \[\hat y_{t,i}^{(s)}\] is the target value (input value at the next time step). Minimizing this negative log-likelihood can be regarded as minimizing the precision-weighted prediction error. Details of feed-forward prediction, activation function, and parameter optimization are described in the Supplementary Methods.

### Experimental procedures and ASD-like performance measures

In the training phase, parameter optimization is performed on the network structure, e.g., synaptic weights, and PB activities corresponding to each target sequence. Target sequences consisted of 6 basic emotions × 16 subjects and were prepared by extracting 9 features from facial expression videos (Supplementary Methods). After training, each target sequence was associated with a specific PB activity, and the relationships (similarities and differences) between target sequences were expected to be “self-organized” in the state space of the PB activity (referred to as “PB space” below). In the test phase, the neural network was required to predict unseen test target sequences. In this test phase, parameter optimization is only applied to PB activities such that the prediction error for test target sequences is minimized while the network structure remains fixed.

This PB update process for the test target sequence was considered “emotion recognition”, based on the similarity of the PB activity for the test sequence to the PB activity clusters for training sequences of the same emotion. This similarity of PB activities within the same emotion is quantitatively evaluated by a clustering index, an average silhouette width, called an “emotion recognition index” herein (Supplementary Methods). In addition to the emotion recognition index, we also included the prediction error of the test target sequence (test error) as an ASD-like performance measure. Based on predictive processing theory, the test error reflects impairments in generalization, a cognitive trait of ASD (Haker et al., 2016). This corresponds to the cognitive tendency in ASD to focus on details of sensory information, making it difficult to extract abstract meaning, resulting in overfitting (Van de Cruys et al., 2013). Eight-fold cross validation was used to calculate ASD-like performance.

### Experimental conditions in neural network simulation

Based on previous studies (Idei et al., 2020; Idei et al., 2021; Lanillos et al., 2020; Takahashi et al., 2021; Thomas et al., 2016), we examined effects of the following two parameters on the ASD-like performance in the neural network model (Figure 1).

The first parameter is the FC_model_, which indicates the proportion of synaptic connections between groups of neurons at different hierarchical levels. FC_model_ is assumed to correspond to FC as measured by fMRI (hereafter referred to as FC_MRI_ in comparison to FC_model_). As shown in Figure 1, the FC_model_ between the PB and the lower-level neuron and the FC_model_ between the lower-level neurons and the input/prediction neurons are referred to as higher-level FC_model_ and lower-level FC_model_, respectively, which were set to one of the following values: 100, 80, 60, 40, or 20%. The second parameter is network excitability homogeneity. The intrinsic homogeneity of network excitability is important for efficient information processing (Hunsberger et al., 2014; Mejias & Longtin, 2012, 2014), and its alterations are thought to be related to ASD, i.e., altered “excitatory-inhibitory balance” (Idei et al., 2020). In the current experiment, as shown in Equation (3), the activity threshold of lower-level neurons, i.e., a_i_ in Equation (1), is initialized to follow a Gaussian distribution and fixed without being updated by learning. Note that in the training phase, the network structure (weight and PB) is optimized based on the distribution of these activity thresholds.

3 \[{{\mathrm{a}}_{\mathrm{i}}}{\sim}{\mathrm{N}}\left({{\mathrm{0,}}\,\,{\mathrm{K}}} \right)\quad \,{\mathrm{K}} = 0.1,{\mathrm{}}1,{\mathrm{}}10\]

The K parameter in Equation (3) determines the homogeneity of intrinsic neuronal excitability, and as parameter K decreases, excitability of the network becomes more homogeneous. In the current analysis, the experimental conditions of K = 0.1, 1, and 10 are referred to as the highly homogeneous, modestly homogeneous, and heterogeneous network conditions, respectively.

### Examining the association between simulation results and fMRI datasets

Based on the assumption that there is a correspondence between neural network parameters and fMRI parameters, we examined whether the relationship between neural network parameters and ASD-like performance in the neural network simulation could be validated between subject subgroups allocated on the basis of fMRI parameters and ASD diagnosis/symptoms in the fMRI dataset. As an fMRI dataset, we used resting state fMRI datasets for 849 subjects (410 ASD and 439 TD) from the publicly available Autism Brain Imaging Data Exchange (Craddock et al., 2013; Di Martino et al., 2014). See Supplementary Methods, Supplementary Table 1, and Supplementary Table 2 for details.

We determined the fMRI parameters corresponding to neural network parameters by the following procedure. First, referring to previous neural circuit studies, insula (Fusar-Poli et al., 2009; Gobbini & Haxby, 2007; Haxby et al., 2002; Wicker et al., 2003; Wright et al., 2004), middle temporal gyrus (temporo-occipital part) (Beauchamp et al., 2004; Deen et al., 2020; Fusar-Poli et al., 2009; Gobbini & Haxby, 2007; Haxby et al., 2002; Sabatinelli et al., 2011; Wang et al., 2016), and visual cortex (intracalcarine cortex) (Bernstein & Yovel, 2015; Fusar-Poli et al., 2009; Haxby et al., 2002) were selected as key anatomical regions for facial emotion recognition, and the three levels of hierarchical neural network model, i.e. PB, lower-level, and input neurons, in the neural network were assumed to correspond to each brain region, respectively. Based on these assumptions, higher-level FC_model_ and lower-level FC_model_ were assumed to correspond to the higher-level FC_MRI_, i.e., FC_MRI_ between insula and middle temporal gyrus, and the lower-level FC_MRI_, i.e., FC_MRI_ between middle temporal gyrus and visual cortex, respectively. In addition, network excitability homogeneity was assumed to correspond to the ReHo of the middle temporal gyrus (Figure 1). It should be noted that there are other choices regarding selection of anatomical regions. For example, other regions related to facial emotion recognition include the prefrontal cortex (7–9), amygdala (7–10), fusiform gyrus (7–9), thalamus (8–10), and parahippocampal gyrus (8, 9). Furthermore, it is sometimes difficult to determine whether a given region serves as a higher-level or lower-level neuron. Application of the simulation results to the fMRI dataset in this study is not intended to be exhaustive and is still at the proof-of-concept stage. The Harvard Oxford atlas was used to identify the above anatomical regions (Makris et al., 2006). The aforementioned FC_MRI_s and ReHo were transformed to normal distributions using Fisher r-z and Box-Cox transformations, respectively, and were adjusted for covariates, i.e., age, sex, FIQ, mean framewise displacement, and sites.

Next, each experimental condition of neural network simulation was mapped to a subject subgroup on the basis of fMRI parameters as follows. In fMRI datasets, 849 subjects were divided into 5 × 5 × 3 = 75 subgroups by dividing them into 5, 5, and 3 groups of approximately the same numbers of subjects, according to their higher/lower-level FC_MRI_, and ReHo ranks, respectively. Each subgroup was assigned to one of 5 × 5 × 3 = 75 neural network parameter settings with higher/lower-level FC_model_ of 20, 40, 60, 80, and 100% and network excitability homogeneity of highly homogeneous, modestly homogeneous, and heterogeneous, respectively.

Finally, we examined whether the subject group corresponding to the experimental condition that exhibited ASD-like performance in the neural network simulation actually had an ASD diagnosis/symptoms.

## Results

### Performance of a typical development (TD) model in neural network simulation

**Figure 2:**
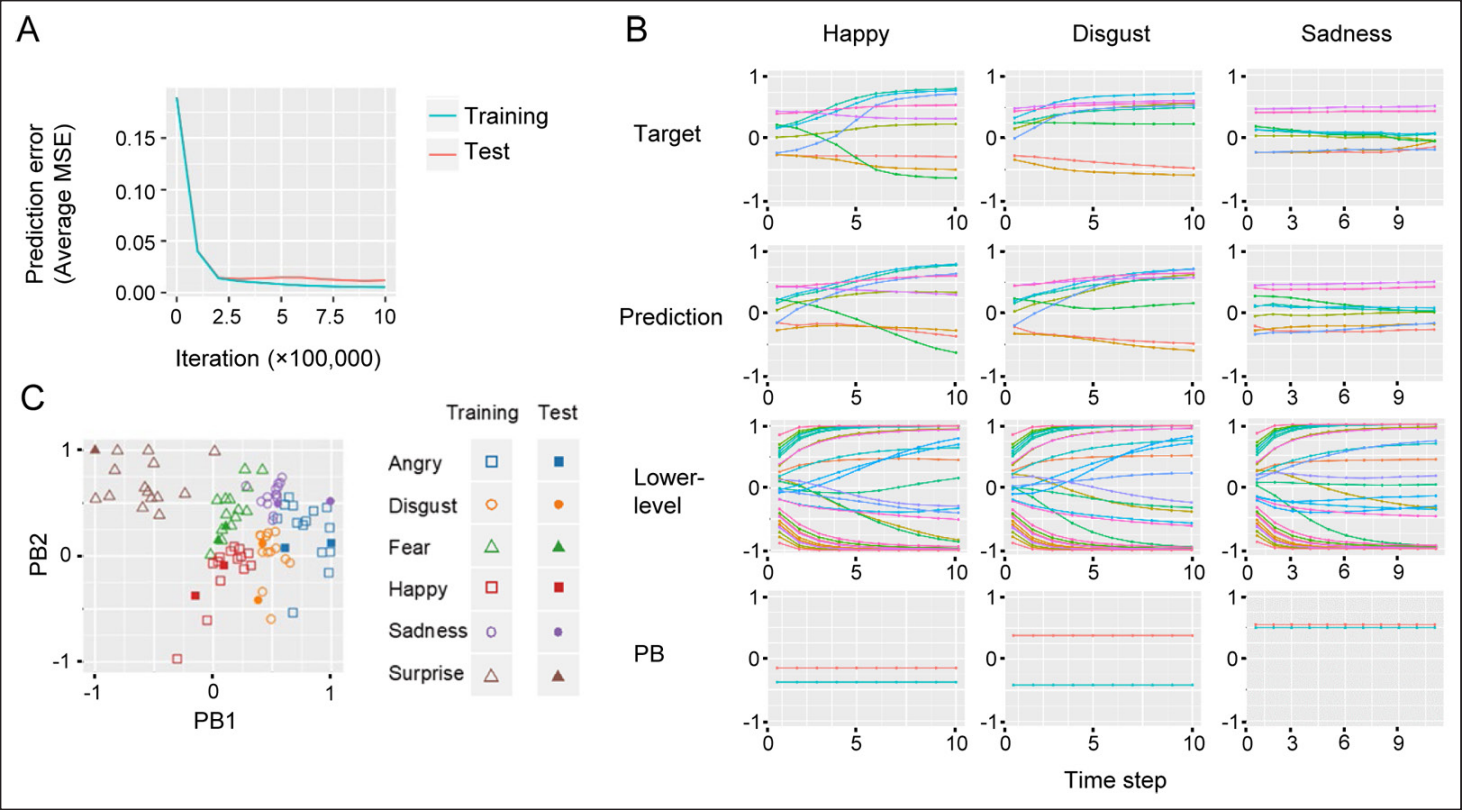
Analytical results for the TD model. **(A)** Learning curves. **(B)** Examples of target, prediction, lower-level neuron, PB activity sequences. **(C)** PB activities corresponding to all target sequences (PB space). *Abbreviation*. MSE, mean squared error; PB, parametric bias.

The network with the experimental condition in which network parameters were set at a heterogeneous network excitability and no reduction in FC, i.e. higher/lower-level FC_model_ = 100%, is called a TD model. The learning curve for the TD model shows that both training and test error substantially decrease (Figure 2A). This indicates that the model not only successfully reproduces the training target sequence, but also generalizes to an unknown test target sequence.

Examples of target, prediction, lower-level neuron and PB activity sequences in the test phase are shown in Figure 2B. The target sequence varies with emotion, and the prediction reproduces the target sequence well. Lower-level neuron activity corresponds to dynamics of the short-term target sequence, whereas PB activity corresponds to a more abstract level of characteristics of the target sequence.

PB activities corresponding to all target sequences, i.e., PB space, are shown in Figure 2C. In Figure 2C, PB activities corresponding to training target sequences seem to form emotion clusters, indicating that emotion clusters are self-organized in the predictive processing framework, even though no emotion labels are given in training. Furthermore, we can see that PB activities corresponding to the test sequence are located near PB clusters of the same emotion as the training sequences. This indicates that the model successfully recognizes facial emotions even for unknown test data based on a predictive processing framework.

### Influence of neural network parameters on ASD-like performance and the interaction between parameters

**Figure 3:**
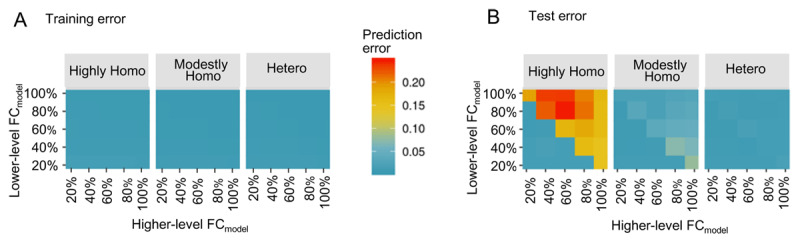
Investigation of prediction error under various experimental conditions **(A)** Training error. **(B)** Test error. *Abbreviations*. Highly Homo, highly homogeneous network condition; modestly Homo, modestly homogeneous network condition; hetero, heterogeneous network condition; FC_model_, functional connectivity in neural network model.

The results of prediction error, one of the ASD-like performance indicators, are shown in Figure 3A (training error) and Figure 3B (test error). These figures show the change in prediction error when varying network excitability homogeneity and the higher/lower-level FC_model_. In the highly homogeneous network with the high FC_model_ condition, the training error was small, but the test error was large, indicating that the model was overfitted to training sequences and failed to acquire generalization capability, i.e., ASD-like performance. On the other hand, in the other network excitability homogeneity conditions (modestly homogeneous and heterogeneous), both training and test errors were small, regardless of the FC_model_ condition, indicating that the model successfully acquired generalization capability to predict emotional facial expressions, i.e., TD-like performance.

**Figure 4:**
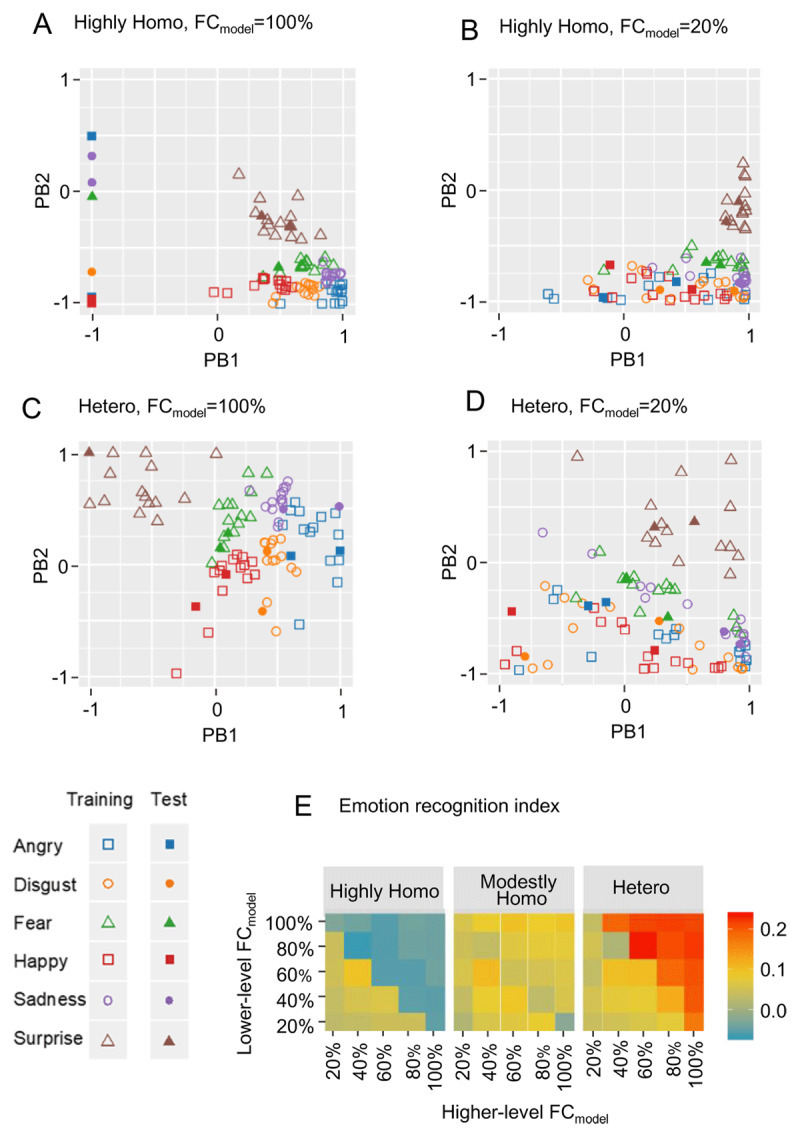
Investigation of emotion recognition performance under various experimental conditions. **(A)(B)(C)(D)** Illustration of PB space when network excitability homogeneity and FC_model_ differ. Note that FC_model_ = 100% and FC_model_ = 20% indicate that both higher-level FC_model_ and lower-level FC_model_ are 100% and 20%, respectively. Experimental conditions in (C) are identical to those in Figure 2(C), and these figures are identical. **(E)** Emotion recognition index. The emotion recognition index, i.e. average silhouette width, is a measure of the similarity between a test PB activity and a training PB activity of the same emotion. See Supplementary Methods for details. *Abbreviations*. Highly Homo, highly homogeneous network condition; modestly Homo, modestly homogeneous network condition; hetero, heterogeneous network condition; FC_model_, functional connectivity in neural network model; PB, parametric bias.

Next, to examine the other ASD-like performance measure, emotion recognition, i.e., the similarity of PB activity for test to training PB activity clusters within the same emotion, we illustrated PB spaces under different experimental conditions (Figure 4A–D and Supplementary Figure 1). Comparing these four PB spaces, in the heterogeneous network with the FC_model_ = 100% (Figure 4C), PB activities for test were most clearly located within training PB activity clusters of the same emotion, i.e., most successful emotion recognition. In the highly homogeneous/heterogeneous network with FC_model_ = 20% (Figure 4B and 4D), PB activities for test were not so far from training PB activity clusters, but boundaries between emotional PB activity clusters seem a little unclear, i.e. almost successful emotion recognition. In the highly homogeneous network with FC_model_ = 100% (Figure 4A), PB activities for test were located farthest away from training PB activity clusters, i.e., most unsuccessful emotional recognition. From these findings, regarding network excitability homogeneity, the homogeneous network condition appears to show more unsuccessful emotion recognition, i.e., ASD-like performance. On the other hand, regarding FC_model_, performance in emotion recognition appears to be reversed, depending on network excitability homogeneity. That is, in a heterogeneous network, the high FC_model_ condition shows more successful emotion recognition, i.e., TD-like performance, than the low FC_model_ condition, while in a highly homogeneous network, the high FC_model_ condition shows more unsuccessful emotion recognition, i.e., ASD-like performance than the low FC_model_ condition. These intuitive findings are more clearly illustrated in Figure 4E by quantitative analyses using a clustering measure of PB activity by emotion called “emotion recognition index”, i.e., average silhouette width, described in Methods. Specifically, regarding network excitability homogeneity, the homogeneous network showed a lower emotion recognition index, i.e., more unsuccessful emotion recognition. Regarding FC_model_, in a heterogeneous network, the high FC condition showed a higher emotion recognition index than the low FC condition, while conversely, in a highly homogeneous network, the high FC condition showed lower emotion recognition index than the low FC condition.

From the above analysis, we found that the two ASD-like performance measures, test error and emotion recognition, share common trends. First, ASD-like performance is exacerbated as network excitability becomes more homogeneous. Second, the effect of FC_model_ on ASD-like performance depends on network excitability homogeneity, i.e., FC_model_ and network excitability homogeneity interact.

### The mechanism by which changes in neural network parameter cause ASD-like performance

In order to clarify the mechanism by which network excitability homogeneity and FC_model_ interact to cause ASD-like performance, we examined how each parameter affects neural representations obtained in developmental learning.

**Figure 5:**
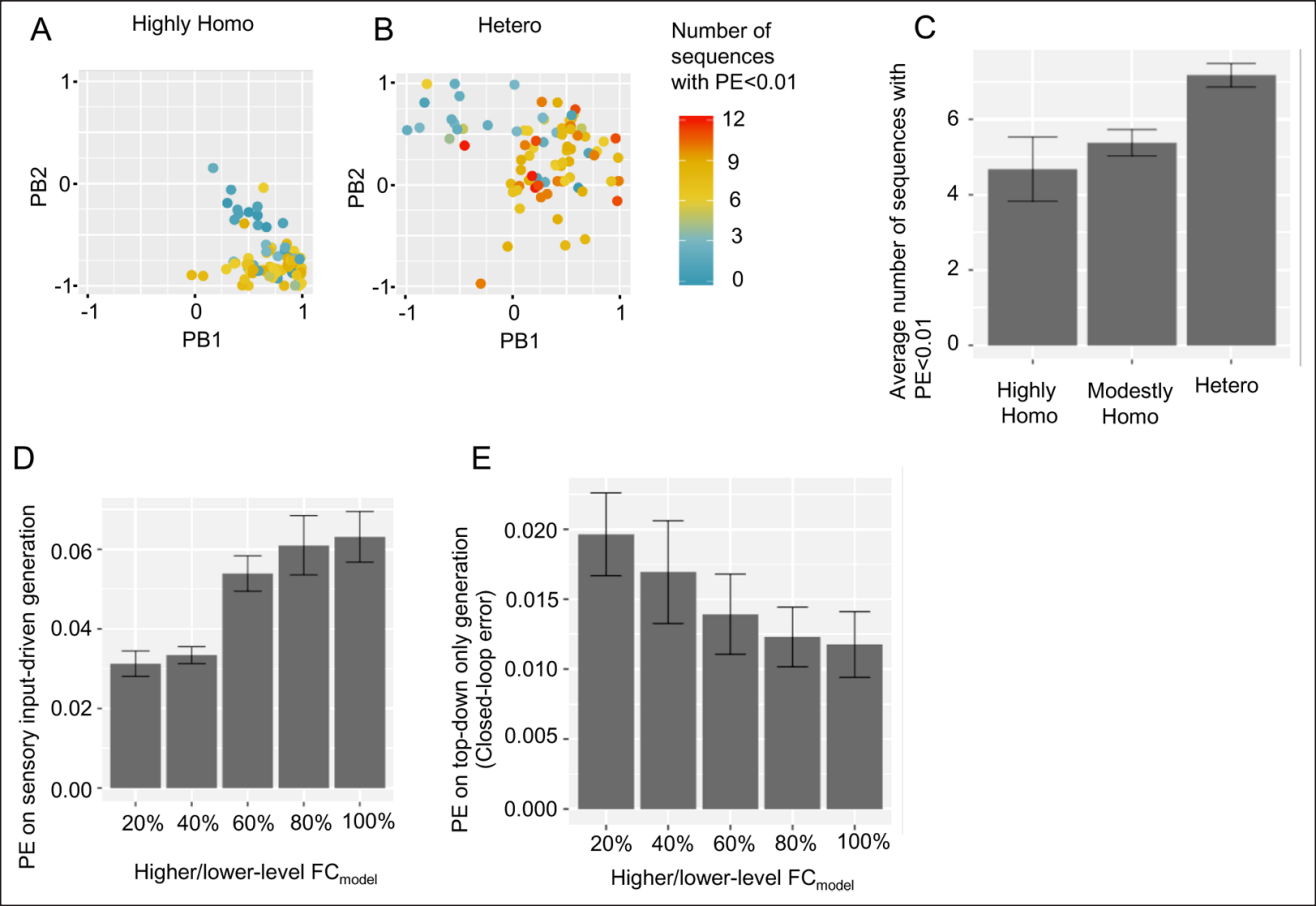
Mechanisms by which underlying ASD-like performance induced by changes in neural network parameters caused ASD-like performance. **(A)(B)** Example of a scatterplot of the tolerance of PB space. Positions of points in the scatterplot represents the PB activity obtained by training, as shown in Figures 3C and 3E. Colors of dots indicate the number of training target sequences that can be predicted with low error (PE < 0.01) by providing that PB activity. Both higher-level and lower-level FC_model_ were set to 100%. **(C)** Bar chart of the average number of training target sequences that can be reproduced with small error (PE < 0.01). **(D)** Prediction error on sensory input-driven generation by setting unreliable (random) PBs. **(E)** Prediction error on top-down only generation by closed-loop generation. *Abbreviations*. Highly Homo, highly homogeneous network condition; modestly Homo, modestly homogeneous network condition; hetero, heterogeneous network condition; PB, parametric bias; PE, prediction error; FC_model_, connectivity proportion.

Regarding network excitability homogeneity, the aforementioned analysis revealed that heterogeneous network conditions tended to acquire generalization capability in that they had lower test error and were more successful in emotion recognition. We then hypothesized that the PB space in a heterogeneous network condition would tolerate subtle differences among target sequences for prediction, while the homogeneous network would be fragile to subtle differences due to overfitting to a particular sequence of the training sequences. To investigate this hypothesis, we evaluated the number of training sequences that could be well predicted, i.e., average mean squared error <0.005, based on a particular value of PB activity, i.e., closed loop analysis. See Supplementary Methods for details. This number of successfully predicted sequences would reflect the tolerability of PB space for application to different sequences. As expected, it is clear that individual PB activities in the heterogeneous network condition (Figure 5B) are able to predict more sequences with small errors than PB activities in the highly homogeneous network condition (Figure 5A), and this is confirmed by quantitative analysis, which calculated the average per network condition (Figure 5C).

As mentioned above, network excitability and FC_model_ interact, and network excitability affected the tolerability of PB space, which influenced the generalization of top-down prediction. Based on the fact that prediction in predictive processing system is based on both top-down prediction and bottom-up sensory input, we hypothesize that FC_model_ determines whether the information processing is top-down prediction dependent or sensory input dependent (or hypo-prior (Pellicano & Burr, 2012)). To investigate this hypothesis, prediction errors for the unreliable (random) PBs, i.e., sensory input-driven generation (Figure 5D), and prediction errors for closed-loop generation, i.e., top-down only generation (Figure 5E), were calculated. In the low FC_model_ condition, the prediction error for sensory input-driven generation was small (Figure 5D), but the prediction error based on top-down only generation was large (Figure 5E), indicating that the low FC_model_ condition induced sensory input-dependent information processing, while in the high FC_model_ condition, the opposite was true: top-down prediction-dependent information processing.

The above examination provides the following explanation for the interaction of neural network parameters to exhibit ASD-like performance. Under the homogeneous network condition, PB space is intolerant, and top-down predictions are overfitted to subtle differences among sequences. As such, when top-down prediction is not accurate for test sequences, high FC_model_ conditions with top-down prediction-dependence exhibit more ASD-like performance than low FC_model_ conditions. On the contrary, under a heterogeneous network condition, PB space is tolerant, and the network provides accurate top-down predictions for test sequences. In this case, high FC_model_ conditions with top-down prediction-dependence exhibit more TD-like performance than low FC_model_ conditions.

In addition, there is a debate as to whether ASD information processing is top-down prediction or sensory input-dependent (hypo-prior) (Idei et al., 2018; Karvelis et al., 2018; Lawson et al., 2014; Pellicano & Burr, 2012; Philippsen & Nagai, 2018, 2020). This study suggests that both top-down prediction-dependent (high FC_model_) and sensory input-dependent (low FC_model_) information processing may exhibit outwardly ASD-like performance, i.e., impaired emotion recognition, depending on the network excitability homogeneity.

### Applicability of neural network simulation results to fMRI data

Neural network simulation showed that parameters interacted to cause ASD-like performance, and provided an explanation of information processing mechanisms. Of these findings, the relationship between parameters and ASD-like performance can be examined for biological reproducibility using the following procedure. First, by assuming a correspondence between the neural network parameter, i.e., network excitability homogeneity and FC_model_, and the fMRI parameter, i.e., regional homogeneity and FC_MRI_, each experimental condition of the neural network is mapped to a subject subgroup identified by fMRI parameters (see Methods for details). As in the Methods, it should be noted that a significant simplification in the selection of anatomical regions has been made in this mapping. Second, we examined whether subject subgroups corresponding to neural networks that exhibited ASD-like performance actually showed more ASD diagnoses/symptoms.

**Figure 6:**
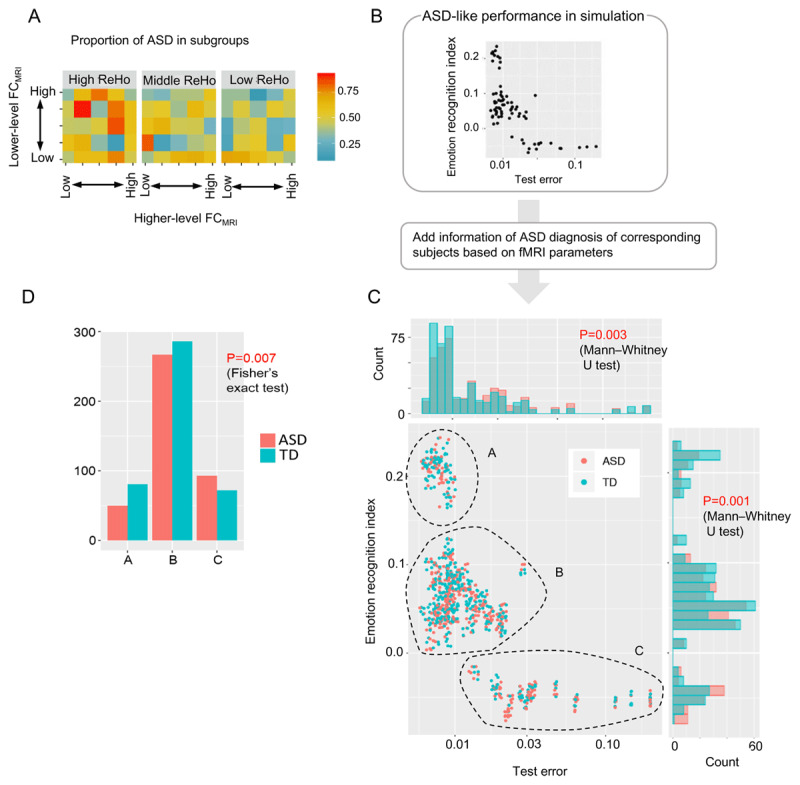
Validation of neural network simulation results using fMRI data. **(A)** Proportion of ASDs belonging to each subgroup. Labels in this figure (fMRI parameters) correspond to labels in Figure 3A, 3B and 3G (neural network parameters). Specifically, neural network parameters, i.e., network excitatory homogeneity and higher/lower-level FC_model_, are mapped to subgroups in fMRI datasets based on fMRI parameters, i.e., regional homogeneity and higher/lower-level FC_model_, respectively. **(B)** Scatterplot of test error and emotion recognition index. **(C)** For Figure (B), the diagnostic information of ASD of the subject corresponding to the neural network of each point is added. (In the scatterplot, a little Gaussian noise is added to each point on the X- and Y-axis for visibility.) Histograms of ASD diagnosis are added about test errors (X-axis) and emotion recognition index (Y-axis). **(D)** Histogram for the number of ASDs and TDs in A, B, and C groups.

Following the above procedure, in order to examine the correspondence between ASD-like performance of neural network and ASD diagnosis in subject subgroup, Figure 6A shows the proportion of ASD diagnoses for each subject subgroup by arranging fMRI parameters for each axis in a way that corresponds to Figure 3B (test error) and Figure 4E (emotion recognition index). Despite variations across subgroups, the trend in ASD diagnosis in the fMRI data set (Figure 6A) appears to be similar to ASD-like performance of neural network simulations (Figures 3B and 4G). Specifically, the overall trend is for ASD diagnoses to be more common in subgroups with higher ReHo (P = 0.021 for the main effect of logistic regression analysis). Furthermore, the impact of FC_MRI_ on ASD diagnosis appears to differ between the high and low ReHo subject groups. That is, in the high ReHo group there seemed to be more ASD patients with high FC_MRI_s, while in the low ReHo group there seemed to be more ASD patients with low FC_MRI_s (P = 0.025 for interaction effect in logistic regression analysis).

To further investigate the correspondence between the ASD-like performance of the neural network and ASD diagnosis in fMRI datasets, the scatterplot of test error and emotion recognition index created from the results of neural network simulation (Figure 6B) was colored to represent the ASD diagnosis of corresponding subjects based on the fMRI parameter (Figure 6C). In the histogram along the X-axis in Figure 6C, there appears to be a statistically significant trend (P = 0.003) toward more ASD among subjects corresponding to neural networks with larger test errors. Next, in the histogram in the Y-axis direction, the subject subgroups corresponding to neural networks with a large emotion recognition index tend to have more TDs, and this is statistically significant (P = 0.001). In the scatter plot in Figure 6C, clusters appear to be divided into the three groups, A, B, and C, shown in the figure, and the number of ASDs and TDs in each of these groups is shown in Figure 6D. There was a significant difference among the three groups (P = 0.007), with more TDs in the group with a high emotion recognition index and low test error (Group A) and more ASDs in the group with a low emotion recognition index and high test error (Group C).

While the above analysis showed an association between the ASD-like performance measure in neural networks and ASD diagnosis in fMRI datasets, we next hypothesized that emotion recognition performance in neural networks would be associated with impaired social interaction symptoms in ASD patients, because emotion recognition is essential for social interaction. To examine this hypothesis, we performed correlation analyses between ASD-like performance measures and each ASD symptom in the ASD population, evaluated by Autism Diagnostic Interview-Revised (ADI-R (Lord et al., 1994)) (Table 1). As we hypothesized, the emotion recognition index was negatively correlated with impaired social interaction symptoms (r = –0.123; 95% CI = –0.233 to –0.008) with a P-value of 0.034. This result suggests similarities between characteristics of neural network performance and corresponding characteristics of subject ASD symptoms.

**Table 1:** Relationship between ASD-like performance of the neural network and ADI-R scores of corresponding subjects.


ADI-R SCALES	NUMBER	CORRELATION COEFFICIENT^a^ WITH TEST ERROR (95% CI)	CORRELATION COEFFICIENT^a^ WITH EMOTION RECOGNITION INDEX (95% CI)

Language/Communication	297	0.014 (–0.105, 0.120)	–0.088 (–0.195, 0.017)

Reciprocal Social Interactions	296	–0.009 (–0.118, 0.092)	–0.123 (–0.233, –0.008)

Restricted, Repetitive, and Stereotyped Behaviors and Interests	296	0.080 (–0.032, 0.206)	–0.111 (–0.225, 0.016)


^a^ Spearman’s correlation coefficient. Before the correlation analysis, mapping from the neural network parameter to the subject subgroup in fMRI datasets is adjusted by covariates, i.e., age, gender, FIQ, mean framewise displacement, and sites. *Abbreviations*. S-CTRNNPB, stochastic continuous time recurrent neural network with parametric bias; ADI-R, Autism Diagnostic Interview-Revised; CI, confidence interval.

## Discussion

In the current study, neural network simulation, which is analogous to the developmental learning process, showed that FC, i.e., FC_model_, and neural excitability, i.e., network excitability homogeneity, interacted to cause ASD-like performance. Behind this interaction, FC determines whether information processing depends on top-down prediction or sensory input, and which of these two types of information processing causes more ASD-like performance depends upon the generalization capability of top-down prediction determined by neural excitability homogeneity. Furthermore, the relationship among FC_model_, neural excitability homogeneity and ASD-like performance in network simulation was biologically validated using fMRI datasets as the relationship among FC_MRI_, ReHo and ASD diagnosis.

In the neural network simulation, by taking an approach that embodies predictive processing theory as system-level neural dynamics, we gained new insights into the relationship among neural excitability, FC, and ASD symptoms. While previous reports have shown that neural excitability alone affected ASD-like performance (Idei et al., 2020; Takahashi et al., 2021), we found that neural excitability not only influences ASD-like performance directly, but also influences the direction of effect of FC, i.e., interacts with FC, with regard to ASD-like performance. While most computational simulation studies to date have examined the relationship between individual parameters, i.e., the strength of prior distribution, and ASD-like performance (Lanillos et al., 2020; Lawson et al., 2017), future research is expected to focus on interactions between multiple parameters.

Although the current study suggests that the interaction between neural excitability and FC underlies ASD symptoms, there are still only a few biological studies that investigate the relationship between these parameters in ASD. Since network excitability homogeneity corresponds to the homogeneity (or synchronization) of neural activities in a local network (Idei et al., 2020; Takahashi et al., 2021), we assumed similarity with ReHo in fMRI datasets (Zang et al., 2004). As another biological approach to examine the relationship between network excitability and FC, there are several studies that measured glutamate/glutamine concentration and FC simultaneously, based on Magnetic Resonance Spectroscopy and fMRI (Hegarty et al., 2018; Siegel-Ramsay et al., 2021). In these studies, the direction of the correlation between neural excitability and FC was reported to differ between ASD and TD (Hegarty et al., 2018; Siegel-Ramsay et al., 2021), which may be due to the interaction between FC and neural excitability, consistent with our findings. These studies are still small pilot studies, and larger biological studies are expected to examine the relationship between neural excitability and FC.

The current study had limitations in that it required a simplification of imaging test results to bridge the gap between simulation studies at an abstract level and complex biological studies. The simulation study used a simple model for clear depiction of the computational theory, but the actual biological brain is of course more complex. Specifically, the biological neural network involved in facial emotion recognition would be formed from a larger number of hierarchies and regions (Fusar-Poli et al., 2009; Sabatinelli et al., 2011; Xu et al., 2021). Since there are multiple brain region candidates for mapping neural networks, bias may occur in this process. To reduce this selection bias, it would be straightforward to perform simulations repeatedly to cover all candidate brain region combinations. However, such an exhaustive simulation here is difficult due to huge computational costs. Another way to reduce the bias while avoiding excessive computer costs may be data-driven approach such as machine learning (Yamagata et al., 2019). However, if the model becomes too complex, it becomes difficult to illustrate information processing in an explainable manner. A possible future direction would be to map imaging data to neural network parameters while avoiding excessive model complexity by using latent variables obtained from unsupervised feature extraction, such as variational autoencoders.

In this study we prepared a static PB. The possibility that this simplification may have affected the final results needs to be discussed. For example, implementing a static PB might have reduced performance in predicting the dynamics of very complex sensory data compared to a model in which the higher-level neurons are dynamic. However, we believe that the impact of static PB settings on the final results is negligible. This is because PBs can bring functional hierarchy to neural networks in the same way as dynamic neurons. Functional hierarchy has been shown to emerge by setting multiple timescale property in neural network (Yamashita & Tani, 2008). And static PBs have been shown to be able to perform as a higher-level neuron similar to that of dynamic higher-level neurons, as a case of infinitely large time constants (Idei et al., 2021; Tani, 2003; Yamashita & Tani, 2012). Furthermore, this multiple time scale property is shown to be present in actual brain activity, as evidenced by recent biological and computational studies (Soltani et al., 2021). Thus, there is biological plausibility in modeling higher-level brain area by neurons with higher time constants.

The fact that the current study design only involved learning and testing once each, may be a limitation in simulating development of ASD. What this study succeeded in showing is that innate parameters (FC and neural excitability homogeneity) affect a single learning session and cause ASD-like performance. However, in the actual developmental process of ASD, learning and testing may be repeated from the beginning of development. As a result of these processes, ASD-specific perceptions and cognition may emerge, as well as compensatory behavioral changes. For example, in ASD, the impact of previous learning on subsequent learning may change during repeated learning (D’Cruz et al., 2013; Schmitt et al., 2019). Future computational modeling research of the developmental process based on repetitive learning should provide a better understanding of ASD.

Other studies have examined the association between complex cognitive process, e.g., emotion recognition, and fMRI using models of underlying computation, e.g., reinforcement learning and Bayesian inference (Cohen et al., 2017; Gläscher & O’Doherty, 2010). A number of these studies have examined the relationship between hidden variable information obtained from the model and specific brain regions (Gläscher & O’Doherty, 2010). However, while reinforcement learning and Bayesian inference can be expressed with few parameters and easy to find corresponding brain regions, it has been increasingly recognized that neural network may be better suited to adequately explain complex cognitive or learning processes (Dezfouli et al., 2019; Idei et al., 2021; Yang et al., 2019). Nevertheless, the framework for biological validation of neural network models has only just begun to be explored. For example, recently, the similarity of the representation between the hidden layers of the Deep Q network and fMRI of healthy subjects has been examined during a video game task (Cross et al., 2021). Compared with these previous studies, the current neural network approach is novel and significant in that it prepares a subject-specific neural network to clarify the correspondence between the model and the original subjects, and uses a model that can examine the developmental learning process behind the emergence of cognitive alterations in ASD.

## Additional Files

The additional files for this article can be found as follows:

10.5334/cpsy.93.s1 Supplementary File 1. Supplementary Methods.

10.5334/cpsy.93.s2 Supplementary File 2. Supplementary Tables 1 and 2.

10.5334/cpsy.93.s3 Supplementary File 3. Supplementary Figure 1.

## References

[1] Beauchamp, M. S., Lee, K. E., Argall, B. D., & Martin, A. (2004). Integration of Auditory and Visual Information about Objects in Superior Temporal Sulcus. Neuron, 41(5), 809–823. DOI: 10.1016/S0896-6273(04)00070-4 15003179

[2] Bernstein, M., & Yovel, G. (2015). Two neural pathways of face processing: A critical evaluation of current models. Neurosci Biobehav Rev, 55, 536–546. DOI: 10.1016/j.neubiorev.2015.06.010 26067903

[3] Cohen, J. D., Daw, N., Engelhardt, B., Hasson, U., Li, K., Niv, Y., Norman, K. A., Pillow, J., Ramadge, P. J., Turk-Browne, N. B., & Willke, T. L. (2017). Computational approaches to fMRI analysis. Nature neuroscience, 20(3), 304–313. DOI: 10.1038/nn.4499 28230848 PMC5457304

[4] Craddock, C., Benhajali, Y., Chu, C., Chouinard, F., Evans, A., Jakab, A., Khundrakpam, B. S., Lewis, J. D., Li, Q., & Milham, M. (2013). The neuro bureau preprocessing initiative: open sharing of preprocessed neuroimaging data and derivatives. Frontiers in Neuroinformatics, 7. DOI: 10.3389/conf.fninf.2013.09.0004123717278

[5] Cross, L., Cockburn, J., Yue, Y., & O’Doherty, J. P. (2021). Using deep reinforcement learning to reveal how the brain encodes abstract state-space representations in high-dimensional environments. Neuron, 109(4), 724–738.e727. DOI: 10.1016/j.neuron.2020.11.021 33326755 PMC7897245

[6] D’Cruz, A.-M., Ragozzino, M. E., Mosconi, M. W., Shrestha, S., Cook, E. H., & Sweeney, J. A. (2013). Reduced behavioral flexibility in autism spectrum disorders. Neuropsychology, 27, 152–160. DOI: 10.1037/a0031721 23527643 PMC3740947

[7] Deen, B., Saxe, R., & Kanwisher, N. (2020). Processing communicative facial and vocal cues in the superior temporal sulcus. NeuroImage, 221, 117191. DOI: 10.1016/j.neuroimage.2020.117191 32711066

[8] Dezfouli, A., Griffiths, K., Ramos, F., Dayan, P., & Balleine, B. W. (2019). Models that learn how humans learn: The case of decision-making and its disorders. PLoS Comput Biol, 15(6), e1006903. DOI: 10.1371/journal.pcbi.1006903 31185008 PMC6588260

[9] Di Martino, A., Yan, C. G., Li, Q., Denio, E., Castellanos, F. X., Alaerts, K., Anderson, J. S., Assaf, M., Bookheimer, S. Y., Dapretto, M., Deen, B., Delmonte, S., Dinstein, I., Ertl-Wagner, B., Fair, D. A., Gallagher, L., Kennedy, D. P., Keown, C. L., Keysers, C., … Milham, M. P. (2014). The autism brain imaging data exchange: towards a large-scale evaluation of the intrinsic brain architecture in autism. Molecular psychiatry, 19(6), 659–667. DOI: 10.1038/mp.2013.78 23774715 PMC4162310

[10] Dickinson, A., Jones, M., & Milne, E. (2016). Measuring neural excitation and inhibition in autism: Different approaches, different findings and different interpretations. Brain research, 1648, 277–289. DOI: 10.1016/j.brainres.2016.07.011 27421181

[11] Friston, K. J., Harrison, L., & Penny, W. (2003). Dynamic causal modelling. NeuroImage, 19(4), 1273–1302. DOI: 10.1016/S1053-8119(03)00202-7 12948688

[12] Friston, K. J., Stephan, K. E., Montague, R., & Dolan, R. J. (2014). Computational psychiatry: the brain as a phantastic organ. Lancet Psychiatry, 1(2), 148–158. DOI: 10.1016/S2215-0366(14)70275-5 26360579

[13] Fusar-Poli, P., Placentino, A., Carletti, F., Landi, P., Allen, P., Surguladze, S., Benedetti, F., Abbamonte, M., Gasparotti, R., Barale, F., Perez, J., McGuire, P., & Politi, P. (2009). Functional atlas of emotional faces processing: a voxel-based meta-analysis of 105 functional magnetic resonance imaging studies. Journal of psychiatry & neuroscience: JPN, 34(6), 418–432. https://pubmed.ncbi.nlm.nih.gov/19949718. https://www.ncbi.nlm.nih.gov/pmc/articles/PMC2783433/19949718 PMC2783433

[14] Gläscher, J. P., & O’Doherty, J. P. (2010). Model-based approaches to neuroimaging: combining reinforcement learning theory with fMRI data. WIREs Cognitive Science, 1(4), 501–510. DOI: 10.1002/wcs.57 26271497

[15] Gobbini, M. I., & Haxby, J. V. (2007). Neural systems for recognition of familiar faces. Neuropsychologia, 45(1), 32–41. DOI: 10.1016/j.neuropsychologia.2006.04.015 16797608

[16] Haker, H., Schneebeli, M., & Stephan, K. E. (2016). Can Bayesian theories of autism spectrum disorder help improve clinical practice? Frontiers in psychiatry, 7, 107. https://www.ncbi.nlm.nih.gov/pmc/articles/PMC4911361/pdf/fpsyt-07-00107.pdf. DOI: 10.3389/fpsyt.2016.00107 27378955 PMC4911361

[17] Haxby, J. V., Hoffman, E. A., & Gobbini, M. I. (2002). Human neural systems for face recognition and social communication. Biological psychiatry, 51(1), 59–67. DOI: 10.1016/S0006-3223(01)01330-0 11801231

[18] Hegarty, J. P., Weber, D. J., Cirstea, C. M., & Beversdorf, D. Q. (2018). Cerebro-Cerebellar Functional Connectivity is Associated with Cerebellar Excitation–Inhibition Balance in Autism Spectrum Disorder. Journal of Autism and Developmental Disorders, 48(10), 3460–3473. DOI: 10.1007/s10803-018-3613-y 29796960

[19] Hull, J. V., Dokovna, L. B., Jacokes, Z. J., Torgerson, C. M., Irimia, A., & Van Horn, J. D. (2017). Resting-State Functional Connectivity in Autism Spectrum Disorders: A Review [Review]. Frontiers in psychiatry, 7(205). DOI: 10.3389/fpsyt.2016.00205 PMC5209637 28101064

[20] Hunsberger, E., Scott, M., & Eliasmith, C. (2014). The competing benefits of noise and heterogeneity in neural coding. Neural Comput, 26(8), 1600–1623. DOI: 10.1162/NECO_a_00621 24877735

[21] Idei, H., Murata, S., Chen, Y., Yamashita, Y., Tani, J., & Ogata, T. (2018). A Neurorobotics Simulation of Autistic Behavior Induced by Unusual Sensory Precision. Computational Psychiatry, 1–19. 30627669 10.1162/cpsy_a_00019 PMC6317752

[22] Idei, H., Murata, S., Yamashita, Y., & Ogata, T. (2020). Homogeneous Intrinsic Neuronal Excitability Induces Overfitting to Sensory Noise: A Robot Model of Neurodevelopmental Disorder. Front Psychiatry, 11, 762. DOI: 10.3389/fpsyt.2020.00762 32903328 PMC7434834

[23] Idei, H., Murata, S., Yamashita, Y., & Ogata, T. (2021). Paradoxical sensory reactivity induced by functional disconnection in a robot model of neurodevelopmental disorder. Neural Networks, 138, 150–163. DOI: 10.1016/j.neunet.2021.01.033 33652371

[24] Itahashi, T., Yamada, T., Watanabe, H., Nakamura, M., Jimbo, D., Shioda, S., Toriizuka, K., Kato, N., & Hashimoto, R. (2014). Altered network topologies and hub organization in adults with autism: a resting-state fMRI study. PLoS One, 9(4), e94115. DOI: 10.1371/journal.pone.0094115 24714805 PMC3979738

[25] Karvelis, P., Seitz, A. R., Lawrie, S. M., & Seriès, P. (2018). Autistic traits, but not schizotypy, predict increased weighting of sensory information in Bayesian visual integration. Elife, 7, e34115. DOI: 10.7554/eLife.34115.032 29757142 PMC5966274

[26] Lanillos, P., Oliva, D., Philippsen, A., Yamashita, Y., Nagai, Y., & Cheng, G. (2020). A review on neural network models of schizophrenia and autism spectrum disorder. Neural Networks, 122, 338–363. DOI: 10.1016/j.neunet.2019.10.014 31760370

[27] Lawson, R. P., Mathys, C., & Rees, G. (2017). Adults with autism overestimate the volatility of the sensory environment. Nature neuroscience, 20(9), 1293. DOI: 10.1038/nn.4615 28758996 PMC5578436

[28] Lawson, R. P., Rees, G., & Friston, K. J. (2014). An aberrant precision account of autism. Front Hum Neurosci, 8, 302. DOI: 10.3389/fnhum.2014.00302 24860482 PMC4030191

[29] Lord, C., Rutter, M., & Le Couteur, A. (1994). Autism Diagnostic Interview-Revised: A revised version of a diagnostic interview for caregivers of individuals with possible pervasive developmental disorders. Journal of Autism and Developmental Disorders, 24(5), 659–685. DOI: 10.1007/BF02172145 7814313

[30] Makris, N., Goldstein, J. M., Kennedy, D., Hodge, S. M., Caviness, V. S., Faraone, S. V., Tsuang, M. T., & Seidman, L. J. (2006). Decreased volume of left and total anterior insular lobule in schizophrenia. Schizophrenia Research, 83(2), 155–171. DOI: 10.1016/j.schres.2005.11.020 16448806

[31] Mejias, J. F., & Longtin, A. (2012). Optimal Heterogeneity for Coding in Spiking Neural Networks. Physical Review Letters, 108(22), 228102. DOI: 10.1103/PhysRevLett.108.228102 23003656

[32] Mejias, J. F., & Longtin, A. (2014). Differential effects of excitatory and inhibitory heterogeneity on the gain and asynchronous state of sparse cortical networks. Front Comput Neurosci, 8, 107. DOI: 10.3389/fncom.2014.00107 25309409 PMC4162374

[33] Murata, S., Namikawa, J., Arie, H., Sugano, S., & Tani, J. (2013). Learning to reproduce fluctuating time series by inferring their time-dependent stochastic properties: Application in robot learning via tutoring. IEEE Transactions on Autonomous Mental Development, 5(4), 298–310. DOI: 10.1109/TAMD.2013.2258019

[34] Murata, S., Yamashita, Y., Arie, H., Ogata, T., Sugano, S., & Tani, J. (2017). Learning to Perceive the World as Probabilistic or Deterministic via Interaction With Others: A Neuro-Robotics Experiment. IEEE Trans Neural Netw Learn Syst, 28(4), 830–848. DOI: 10.1109/TNNLS.2015.2492140 26595928

[35] Pellicano, E., & Burr, D. (2012). When the world becomes ‘too real’: a Bayesian explanation of autistic perception. Trends Cogn Sci, 16(10), 504–510. DOI: 10.1016/j.tics.2012.08.009 22959875

[36] Philippsen, A., & Nagai, Y. (2018). Understanding the cognitive mechanisms underlying autistic behavior: a recurrent neural network study. 2018 Joint IEEE 8th International Conference on Development and Learning and Epigenetic Robotics (ICDL-EpiRob). DOI: 10.1109/DEVLRN.2018.8761038

[37] Philippsen, A., & Nagai, Y. (2020). Deficits in Prediction Ability Trigger Asymmetries in Behavior and Internal Representation [Review]. Frontiers in psychiatry, 11(1253). DOI: 10.3389/fpsyt.2020.564415 PMC7716881 33329104

[38] Sabatinelli, D., Fortune, E. E., Li, Q., Siddiqui, A., Krafft, C., Oliver, W. T., Beck, S., & Jeffries, J. (2011). Emotional perception: Meta-analyses of face and natural scene processing. NeuroImage, 54(3), 2524–2533. DOI: 10.1016/j.neuroimage.2010.10.011 20951215

[39] Schmitt, L. M., Bojanek, E., White, S. P., Ragozzino, M. E., Cook, E. H., Sweeney, J. A., & Mosconi, M. W. (2019). Familiality of behavioral flexibility and response inhibition deficits in autism spectrum disorder (ASD). Mol Autism, 10(1), 47. DOI: 10.1186/s13229-019-0296-y 31857874 PMC6909569

[40] Siegel-Ramsay, J. E., Romaniuk, L., Whalley, H. C., Roberts, N., Branigan, H., Stanfield, A. C., Lawrie, S. M., & Dauvermann, M. R. (2021). Glutamate and functional connectivity - support for the excitatory-inhibitory imbalance hypothesis in autism spectrum disorders. Psychiatry Research: Neuroimaging, 313, 111302. DOI: 10.1016/j.pscychresns.2021.111302 34030047

[41] Soltani, A., Murray, J. D., Seo, H., & Lee, D. (2021). Timescales of cognition in the brain. Current Opinion in Behavioral Sciences, 41, 30–37. DOI: 10.1016/j.cobeha.2021.03.003 34026949 PMC8136243

[42] Takahashi, Y., Murata, S., Idei, H., Tomita, H., & Yamashita, Y. (2021). Neural network modeling of altered facial expression recognition in autism spectrum disorders based on predictive processing framework. Scientific reports, 11(1), 14684. DOI: 10.1038/s41598-021-94067-x 34312400 PMC8313712

[43] Tani, J. (2003). Learning to generate articulated behavior through the bottom-up and the top-down interaction processes. Neural Networks, 16(1), 11–23. https://ac.els-cdn.com/S0893608002002149/1-s2.0-S0893608002002149-main.pdf?_tid=46e48d72-2961-4e73-ba9b-d0ddaf28335b&acdnat=1536928184_04fc37ef6c653143d88a9ddeadfd1536. DOI: 10.1016/S0893-6080(02)00214-9 12576102

[44] Thomas, M. S., Davis, R., Karmiloff-Smith, A., Knowland, V. C., & Charman, T. (2016). The over-pruning hypothesis of autism. Developmental Science, 19(2), 284–305. DOI: 10.1111/desc.12303 25845529

[45] Van de Cruys, S., de-Wit, L., Evers, K., Boets, B., & Wagemans, J. (2013). Weak Priors versus Overfitting of Predictions in Autism: Reply to Pellicano and Burr (TICS, 2012). i-Perception, 4(2), 95–97. DOI: 10.1068/i0580ic 23755353 PMC3677336

[46] Vasa, R. A., Mostofsky, S. H., & Ewen, J. B. (2016). The Disrupted Connectivity Hypothesis of Autism Spectrum Disorders: Time for the Next Phase in Research. Biol Psychiatry Cogn Neurosci Neuroimaging, 1(3), 245–252. DOI: 10.1016/j.bpsc.2016.02.003 28083565 PMC5222574

[47] Wang, X., Song, Y., Zhen, Z., & Liu, J. (2016). Functional integration of the posterior superior temporal sulcus correlates with facial expression recognition. Hum Brain Mapp, 37(5), 1930–1940. DOI: 10.1002/hbm.23145 26915331 PMC6867343

[48] Wicker, B., Keysers, C., Plailly, J., Royet, J.-P., Gallese, V., & Rizzolatti, G. (2003). Both of Us Disgusted in My Insula: The Common Neural Basis of Seeing and Feeling Disgust. Neuron, 40(3), 655–664. DOI: 10.1016/S0896-6273(03)00679-2 14642287

[49] Wright, P., He, G., Shapira, N. A., Goodman, W. K., & Liu, Y. (2004). Disgust and the insula: fMRI responses to pictures of mutilation and contamination. NeuroReport, 15(15), 2347–2351. DOI: 10.1097/00001756-200410250-00009 15640753

[50] Xu, P., Peng, S., Luo, Y. J., & Gong, G. (2021). Facial expression recognition: A meta-analytic review of theoretical models and neuroimaging evidence. Neurosci Biobehav Rev, 127, 820–836. DOI: 10.1016/j.neubiorev.2021.05.023 34052280

[51] Yamagata, B., Itahashi, T., Fujino, J., Ohta, H., Nakamura, M., Kato, N., Mimura, M., Hashimoto, R.-i., & Aoki, Y. (2019). Machine learning approach to identify a resting-state functional connectivity pattern serving as an endophenotype of autism spectrum disorder. Brain imaging and behavior, 13(6), 1689–1698. DOI: 10.1007/s11682-018-9973-2 30280304

[52] Yamashita, Y., & Tani, J. (2008). Emergence of Functional Hierarchy in a Multiple Timescale Neural Network Model: A Humanoid Robot Experiment. PLOS Computational Biology, 4(11), e1000220. DOI: 10.1371/journal.pcbi.1000220 18989398 PMC2570613

[53] Yamashita, Y., & Tani, J. (2012). Spontaneous prediction error generation in schizophrenia. PLoS One, 7(5), e37843. DOI: 10.1371/journal.pone.0037843 22666398 PMC3364276

[54] Yang, G. R., Joglekar, M. R., Song, H. F., Newsome, W. T., & Wang, X. J. (2019). Task representations in neural networks trained to perform many cognitive tasks. Nat Neurosci, 22(2), 297–306. DOI: 10.1038/s41593-018-0310-2 30643294 PMC11549734

[55] Zang, Y., Jiang, T., Lu, Y., He, Y., & Tian, L. (2004). Regional homogeneity approach to fMRI data analysis. NeuroImage, 22(1), 394–400. DOI: 10.1016/j.neuroimage.2003.12.030 15110032

