# Supplementary material for: Interaction between Functional Connectivity and Neural Excitability in Autism: A Novel Framework for Computational Modeling and Application to Biological Data
Source: Comput Psychiatr. 2023 Jan 20;7(1):14–29. doi: 10.5334/cpsy.93 (PMC11104370; doi:10.5334/cpsy.93)
Supplement: Supplementary File 1. — Supplementary Methods. [file cpsy-7-1-93-s1.pdf]

Supplementary Methods

Feed-forward prediction

The internal state of the ith neuron at time step t for the sth sequence in the stochastic continuous time recurrent neural network with parametric bias (S-CTRNNPB) is calculated as follows.

$$u_{t,i}^{(s)} = \begin{cases} u_{t-1,i}^{(s)} & i \in I_P \\ \frac{1}{\tau_i}\left(\sum_{j \in I_I} w_{ij} x_{t,j}^{(s)} + \sum_{j \in I_L} w_{ij} l_{t-1,j}^{(s)} + \sum_{j \in I_P} w_{ij} p_{t,j}^{(s)} + a_i\right) + \left(1 - \frac{1}{\tau_i}\right) u_{t-1,i}^{(s)} & i \in I_L \\ \sum_{j \in I_L} w_{ij} l_{t,j}^{(s)} + a_i & i \in I_M, I_V \end{cases} \quad (1)$$

where I_I, I_P, I_L, I_M, and I_V are the index sets of the input, parametric bias (PB), lower-level, predicted mean, and variance neurons, respectively; w_{ij} is the synaptic connection weight from the jth neuron to the ith neuron; $x_{t,j}^{(s)}$ is the jth external input value at time step t of the sth sequence, $l_{t,j}^{(s)}$ is the jth lower-level neuron, $p_{t,j}^{(s)}$ is the jth PB activity; τ_i is the time constant of the ith neuron; and a_i is the activity threshold of the ith neuron.

As represented in the above equation, S-CTRNNPB is a hierarchical neural network model, and a higher-level neuron is called PB. Equation (1) indicates that the internal state of PB neurons does not change through time. In addition, the term $\sum_{j \in I_L} w_{ij} l_{t-1,j}^{(s)}$ (i $\in I_L$) corresponds to the weighted sum of the activity from other lower-level neurons in the previous time step, i.e., recurrent input, and this model is a

recurrent neural network. Also, the term $\left(1-\frac{1}{\tau_i}\right)u_{t-1,i}^{(s)}$ (i $\in I_L$) indicates that the lower-levelneurons preserve their own previous state, which constitutes a leaky integrator unit.

The output of each neuron is calculated by the activation function as follows.

$$p_{t,i}^{(s)} = \tanh\left(u_{t,i}^{(s)}\right) \qquad 1 \le t \cap i \in I_P \tag{2}$$

$$l_{t,i}^{(s)} = \tanh\left(u_{t,i}^{(s)}\right) \qquad 0 \le t \cap i \in I_L \tag{3}$$

$$y_{t,i}^{(s)} = \tanh\left(u_{t,i}^{(s)}\right) \qquad 1 \le t \cap i \in I_M \tag{4}$$

$$v_{t,i}^{(s)} = \exp\left(u_{t,i}^{(s)}\right) \qquad 1 \le t \cap i \in I_V \tag{5}$$

Parameter optimization

S-CTRNNPB uses the gradient descent method for parameter optimization, striving to minimize the negative log-likelihood that assumes a Gaussian distribution for observations, as shown in the following equation.

$$L_{t,i}^{(s)} = \frac{\ln\left(2\pi v_{t,i}^{(s)}\right)}{2} + \frac{\left(\hat{y}_{t,i}^{(s)} - y_{t,i}^{(s)}\right)^2}{2v_{t,i}^{(s)}} \tag{6}$$

where $\hat{y}_{t,i}^{(s)}$ is the target value. Minimizing this negative log likelihood can be viewed as minimizing the precision-weighted (inverse variance-weighted) prediction error.

Parameter optimization is performed by minimizing the sum of the negative log-likelihoods for all feature dimensions, time steps, and sequences, as follows.

$$L = \sum_{s \in I_S} \sum_{t=1}^{T^{(s)}} \sum_{i \in I_M} L_{t,i}^{(s)}, \tag{7}$$

where I_S and $T^{(s)}$ denote the index set and length of the sth target sequence, respectively. The partial derivative of each parameter, $(\partial L)/(\partial \theta)$, can be solved using the back-propagation-through-time method [1].

In both the training and testing phases, parameters that may be optimized are collected by θ, and θ in the nth iteration is updated by gradient descent on the accumulated negative log-likelihood L.

$$\theta(n) = \theta(n-1) + \Delta\theta(n) \tag{8}$$

$$\Delta\theta(n) = -\alpha \frac{\partial L}{\partial \theta} + \eta \Delta\theta(n-1) \tag{9}$$

Here, α is the learning rate and η is the coefficient representing the momentum term. In the current study, α was set to 0.001 and η to 0.9.

In the training phase, synaptic weights, activity thresholds of predicted mean and variance neurons, and PB activity were updated 1,000,000 times, and in the testing phase, only PB activity was updated (100 times). The network was trained to predict future input values with a time delay ζ (ζ = 3 in the experiment) by receiving the input state $x_{t,j}^{(s)}$ at the current time step t. Therefore, the target value in Equation (6) satisfies $\hat{y}_{t,i} = x_{t+\zeta,j}^{(s)}$.

Parameter setting

The numbers of input, predicted mean, and predicted variance neurons are N_{I} =N_{M} = N_{V} = 9, respectively, corresponding to the dimension of the target sequence, and the number of PB neurons is N_{P} = 2. The number of lower-level neurons and time constants are N_{C} = 30 and τ_i = 5, respectively. Note that each neuron in S-CTRNNPB, models the firing frequency of a population of neurons, not the activity of individual neurons in biological brain. During the training phase, weights of the synaptic connections w_{ij} $(j \in I_I, I_L)$ were initialized with random values that follow a uniform distribution on the intervals $\left[-\frac{1}{N_I}, \frac{1}{N_I}\right]$ $(j \in I_I,)$ and $\left[-\frac{1}{N_L}, \frac{1}{N_L}\right]$ $(j \in I_L,)$. Activity thresholds of mean predicted and variance neurons a_i $(i \in I_M, I_V)$ of the mean and variance neurons were initialized with random values that follow a uniform distribution on the intervals [-1,1]. The internal states of PB neurons were initialized to 0 before the training phase.

Assessment of the ability to reproduce the training target sequence (training error and closed loop analysis)

Before proceeding from the training to the test phase, we evaluated the performance of the trained model in reproducing the training target sequences. First, to evaluate the reproduction performance based on both sensory information and the internal model, we used the training target sequence as input, set the corresponding PB, and evaluated the

prediction error (called “training error”). Second, to evaluate the performance of reproducing the target sequence based on the sensory information only, we used the training target sequence as input, set the random (unreliable) PB, and evaluated the prediction error. Third, to evaluate the performance of reproducing the target sequence based on the internal model only, we evaluated the ability to reproduce the sequence in isolation from the external environment. In this analysis, only the initial time step of the training target sequence was given as input, and the corresponding PB was set. The sequence was then reproduced using the sensory states predicted by itself in one time step as input in the next time step. This analysis is called "closed loop" analysis.

Preparation of target sequence

Facial expression videos were obtained from the CK+ public database [2, 3] and include videos of the face changing from neutral to peak emotion. Written informed consent was obtained for the analysis and publication of the images. These movies consist of image frames taken 30 times per second. Each movie in the CK+ database is labeled with an emotion based on criteria related to the movement of facial landmarks, i.e., the Facial Action Coding System [4] and perceptual judgments by several testers [2, 3]. In this study, facial expression movies for six basic emotions (anger, disgust, fear, joy, sadness, and surprise) were used [5].

From the videos, we extracted X-Y coordinates of 68 facial landmarks (136 features) using the automatic face detection and feature tracking system. Then, due to computational cost limitations, features with very strong correlations with other features or features that hardly move were removed, and the remaining 9 features (the X-coordinate of the lip corner, and Y-coordinates of the middle of the eyebrow, the inner eyebrow, ala of the nose, the central upper lip, upper lip vermillion, lip corner, the central lower lip and lower lip vermillion, in the right face) were used for analysis.

Preprocessing of target sequence data was performed using the following Equation (10) and (13). Suppose that we have a sequence of $\boldsymbol{x}^{(i,j)} = \left(x_1^{(i,j)}, x_2^{(i,j)}, x_3^{(i,j)}, \cdots, x_{T^{(i)}}^{(i,j)}\right)$ of the jth feature $(1 \leq j \leq 9)$ in the ith facial expression sequence $(1 \leq i \leq 96)$ with $T^{(i)}$ time step. First, we set the value of the first step of each feature to zero by subtracting its first step value from the vector of sequences.

$$\boldsymbol{x}'^{(i,j)} = \left(0, x_2^{(i,j)} - x_1^{(i,j)}, x_3^{(i,j)} - x_1^{(i,j)}, \cdots, x_{T^{(i)}}^{(i,j)} - x_1^{(i,j)}\right) \quad (10)$$

Next, the values were scaled to a range of values into [-0.9, 0.9] for each feature over all target sequences.

$$MAX = max\left(max_{1\le t\le T^{(1)}}\left(x'^{(1,j)}_{t}\right),\ max_{1\le t\le T^{(2)}}\left(x'^{(2,j)}_{t}\right), \cdots,\ max_{1\le t\le T^{(96)}}\left(x'^{(96,j)}_{t}\right)\right) \tag{11}$$

$$MIN = min\left(min_{1\le t\le T^{(1)}}\left(x'^{(1,j)}_{t}\right),\ min_{1\le t\le T^{(2)}}\left(x'^{(2,j)}_{t}\right), \cdots,\ min_{1\le t\le T^{(96)}}\left(x'^{(96,j)}_{t}\right)\right) \tag{12}$$

$$x''^{(i,j)} = \frac{x'^{(i,j)} - MIN}{MAX - MIN} \times 1.8 - 0.9 \tag{13}$$

The subtraction in equation (10) is the process of unifying the position of features in the first step among all target sequences. This process can be interpreted as a "mapping to normal face normalization," and the previous study has shown that this process is necessary for facial emotion recognition using S-CTRNNPB [6].

Emotion recognition index

The emotion recognition index can be obtained by calculating the clustering index, i.e., silhouette width, in PB space. In calculating the silhouette width, we first define $\mathrm{a}(i)$ for data point $\mathrm{i} \in C_i$ (Ci is the emotion cluster) as follows.

$$\mathrm{a}(i) = \frac{1}{|C_i| - 1} \sum_{j\in C_i, i\neq j} d(i,j) \tag{14}$$

where $|C_i|$ is the number of points belonging to cluster C_i, and $d(i,j)$ is the distance between data points i and j. Then, $\mathrm{a}(i)$ is the average of the distances between i and all other points that belong to the same emotional cluster as i. Therefore, $\mathrm{a}(i)$ can be

interpreted as a measure of how close data point i is to the emotional cluster to which it belongs.

Next, $\mathrm{b}(i)$ is defined as follows.

$$\mathrm{b}(i) = \min_{k \neq i} \frac{1}{|C_k|} \sum_{j \in C_k} d(i, j) \tag{15}$$

Then $\mathrm{b}(i)$ represents the minimum of the average of the distances between i and all points belonging to the other emotion clusters to which i does not belong. Thus, b(i) can be interpreted as a measure of how far away data point i is from the "proximity emotion cluster".

The silhouette width of data point i, $\mathrm{s}(i)$, is calculated as follows.

$$\mathrm{s}(i) = \frac{b(i) - a(i)}{max\{a(i), b(i)\}} \tag{16}$$

Therefore, it is clear that

$$-1 \leq \mathrm{s}(i) \leq 1 \tag{17}$$

Note that the silhouette width ($\mathrm{s}(i)$) becomes larger when the data point i is located farther away from another nearby emotion cluster, i.e., $b(i)$ is larger, and closer to the same emotion cluster, i.e., $a(i)$ is smaller. In the current study, we refer to the average silhouette width (s(i)) where i is the test and j is the training data as the Emotional recognition index. The Emotional recognition index can be interpreted as the performance measure of recognizing the emotion-based similarity and difference

between the unknown test sequence and the trained sequence using PB clusters for each emotion self-organized by developmental learning.

Acquisition and preprocessing of functional magnetic resonance imaging (fMRI) data

For the fMRI data set, we used the Autism Brain Imaging Data Exchange (ABIDE) [7] (http://fcon_1000.projects.nitrc.org/indi/ abide/), a public resting state fMRI data set. In ABIDE, the diagnosis of ASD is provided either by clinical judgement or 'gold standard' diagnostic instruments—Autism Diagnostic Interview Revised (ADI-R) [8] and/or Autism Diagnostic Observation Schedule (ADOS) [9]. As in other ABIDE studies [10-12], inclusion criteria for subjects are as follows. (1) no failures on any of the ABIDE Rater assessments, (2) successful registration with anatomical images covering almost the entire brain, (3) enrollment of at least 10 subjects in both the ASD and TD groups at each center, and (4) no missing covariate data to be used in this study.

Preprocessing of ABIDE was performed by the Preprocessed Connectomes Project (PCP, http://preprocessed-connectomes-project.org/abide/index.html) using the Configurable Pipeline for the Analysis of Connectomes (CPAC) Toolbox [13]. Preprocessing steps included slice timing correction, motion correction, skull-strip, global mean intensity normalization, nuisance signal regression, band-pass filtering

(0.01 - 0.1 Hz), registration to anatomical space, and registration to standard space. See the description in PCP for details (http://preprocessed-connectomes-project.org/abide/cpac.html).

Reference

1. Rumelhart, D.E., G.E. Hinton, and R.J. Williams, *Learning representations by back-propagating errors.* nature, 1986. **323**(6088): p. 533-536.
2. Kanade, T., J.F. Cohn, and Y. Tian. *Comprehensive database for facial expression analysis.* in *Proceedings Fourth IEEE International Conference on Automatic Face and Gesture Recognition (Cat. No. PR00580)*. 2000. IEEE.
3. Lucey, P., et al. *The extended cohn-kanade dataset (ck+): A complete dataset for action unit and emotion-specified expression*. in *2010 ieee computer society conference on computer vision and pattern recognition-workshops*. 2010. IEEE.
4. Ekman, P., W. Friesen, and J. Hager, *Facial action coding system: Research Nexus.* Network Research Information, Salt Lake City, UT, 2002. **1**.
5. Ekman, P., *Facial expressions of emotion: an old controversy and new findings.* Philos Trans R Soc Lond B Biol Sci, 1992. **335**(1273): p. 63-9.
6. Takahashi, Y., et al., *Neural network modeling of altered facial expression recognition in autism spectrum disorders based on predictive processing framework.* Scientific Reports, 2021. **11**(1): p. 14684.
7. Di Martino, A., et al., *The autism brain imaging data exchange: towards a large-scale evaluation of the intrinsic brain architecture in autism.* Molecular Psychiatry, 2014. **19**(6): p. 659-667.
8. Lord, C., M. Rutter, and A. Le Couteur, *Autism Diagnostic Interview-Revised: A revised version of a diagnostic interview for caregivers of individuals with possible pervasive developmental disorders.* Journal of Autism and Developmental Disorders, 1994. **24**(5): p. 659-685.
9. Lord, C., et al., *The Autism Diagnostic Observation Schedule—Generic: A Standard Measure of Social and Communication Deficits Associated with the Spectrum of Autism.* Journal of Autism and Developmental Disorders, 2000. **30**(3): p. 205-223.
10. Hahamy, A., M. Behrmann, and R. Malach, *The idiosyncratic brain: distortion of spontaneous connectivity patterns in autism spectrum disorder.* Nat Neurosci, 2015. **18**(2): p. 302-9.
11. Li, M., et al., *Co-activation patterns across multiple tasks reveal robust anti-correlated functional networks.* NeuroImage, 2021. **227**: p. 117680.
12. Harlalka, V., et al., *Atypical Flexibility in Dynamic Functional Connectivity Quantifies the Severity in Autism Spectrum Disorder.* Frontiers in Human Neuroscience, 2019. **13**(6).
13. Craddock, C., et al., *The neuro bureau preprocessing initiative: open sharing of preprocessed neuroimaging data and derivatives.* Frontiers in Neuroinformatics, 2013. **7**.
