# Supplementary material for: Interaction between Functional Connectivity and Neural Excitability in Autism: A Novel Framework for Computational Modeling and Application to Biological Data
Source: Comput Psychiatr. 2023 Jan 20;7(1):14–29. doi: 10.5334/cpsy.93 (PMC11104370; doi:10.5334/cpsy.93)
Supplement: Supplementary File 2. — Supplementary Tables 1 and 2. [file cpsy-7-1-93-s2.pdf]

Supplementary Table 1. Demographic information of fMRI datasets.

Site	Number		Age					Gender (M/F)		
	ASD	TD	ASD		TD		P[a]	ASD	TD	P[b]
			Mean	SD	Mean	SD				
KKI	16	27	10.4	1.3	10.0	1.2	0.044	12/4	19/8	1.000
LEUVEN_1	14	14	21.9	4.1	23.0	2.8	0.213	14/0	14/0	NA
MAX_MUN	20	28	24.3	13.8	24.6	8.8	0.002	17/3	27/1	0.294
NYU	74	98	14.8	7.1	15.5	6.0	0.252	64/10	73/25	0.058
OHSU	11	13	11.1	1.9	10.2	1.0	0.725	11/0	13/0	NA
OLIN	17	15	16.1	3.1	16.7	3.6	0.581	15/2	13/2	1.000
PITT	25	26	18.4	6.9	18.7	6.7	0.785	22/3	22/4	1.000
SDSU	12	17	14.8	1.8	13.9	1.9	0.980	11/1	11/6	0.187
STANFORD	16	20	9.8	1.6	10.0	1.6	0.116	13/3	16/4	1
TRINITY	22	25	16.8	3.2	17.1	3.8	0.941	22/0	25/0	NA
UCLA_1	41	29	13.1	2.6	13.4	2.2	0.391	35/6	25/4	1
UCLA_2	12	12	12.8	1.9	12.3	1.2	1.000	12/0	10/2	0.478
UM_1	51	50	12.7	2.4	13.8	3.1	0.260	43/8	34/16	0.064
UM_2	13	21	14.9	1.6	16.7	4.0	0.732	12/1	20/1	1.000
USM	45	25	23.2	8.3	21.3	8.4	0.420	45/0	25/0	NA
YALE	21	19	12.9	3.0	13.6	2.1	0.663	13/8	11/8	1.000
Total	410	439	15.8	7.0	15.7	6.2	0.638	361/49	358/81	0.010

Supplementary Table 1 continued.

Site	FIQ					ADI-R social		ADI-R verbal		ADI-R stereo	
	ASD		TD		P[a]	Mean	SD	Mean	SD	Mean	SD
	Mean	SD	Mean	SD							
KKI	93.3	18.4	112.5	9.3	0.063	19.3	5.9	14.8	5.3	5.5	1.5
LEUVEN_1	109.4	13.1	115.4	13.5	0.354	NA	NA	NA	NA	NA	NA
MAX_MUN	109.1	14.2	111.8	11.1	0.268	12.3	3.5	8.8	2.2	3.5	1.3
NYU	107.4	16.4	113.0	16.5	0.424	19.0	5.7	15.8	4.5	5.5	2.6
OHSU	106.0	22.0	114.1	8.9	0.676	19.6	6.7	17.1	5.8	7.4	3.2
OLIN	112.6	18.3	113.9	8.6	0.968	NA	NA	NA	NA	NA	NA
PITT	110.1	15.2	109.5	8.9	0.572	20.8	3.7	16.1	3.6	6.4	2.3
SDSU	114.5	17.3	104.7	8.6	0.010	16.9	5.3	12.7	5.5	6.3	1.8
STANFORD	112.5	14.5	112.1	15.4	0.974	19.6	5.5	14.8	5.0	5.1	2.1
TRINITY	108.9	15.5	110.9	12.2	0.655	20.0	6.2	15.6	5.0	5.4	2.7
UCLA_1	102.8	13.1	104.8	10.0	0.863	20.2	4.8	16.7	4.4	7.4	2.3
UCLA_2	94.2	11.7	111.6	11.9	0.754	20.0	6.5	15.1	5.5	6.6	3.0
UM_1	103.7	17.6	107.0	9.7	0.503	19.8	4.8	15.4	3.8	6.5	2.3
UM_2	114.1	12.9	111.1	9.5	0.576	17.9	5.8	16.2	3.0	6.2	3.3
USM	99.8	16.7	115.4	15.1	0.0628	NA	NA	NA	NA	NA	NA
YALE	93.9	23.4	103.2	15.9	0.138	21.4	6.1	17.9	4.6	5.2	2.8
Total	105.4	17.1	110.7	12.4	7.66×10^{-7}	19.6	5.5	15.7	4.6	6.1	2.6

[a] P-value form Mann–Whitney U test

[b] P-value form Fisher’s exact test

Abbreviations. ASD, autism spectrum disorder; TD, typically developing control; ADI-R social, Reciprocal Social Interaction Subscore Total for Autism Diagnostic Interview-Revised; ADI-R verbal, Abnormalities in Communication Subscore Total for Autism Diagnostic Interview-Revised; ADI-R stereo, Restricted, Repetitive, and Stereotyped Patterns of Behavior Subscore Total for Autism Diagnostic Interview-Revised; KKI, Kennedy Krieger Institute; LEUVEN_1, University of Leuven: Sample 1; MAX_MUN, Ludwig Maximilians University Munich; NYU, NYU Langone Medical Center; OHSU, Oregon Health and Science University; OLIN, Olin, Institute of Living at Hartford Hospital; PITT, University of Pittsburgh School of Medicine; SDSU, San Diego State University; STANFORD, Stanford University; TRINITY, Trinity Centre for Health Sciences; UCLA_1, University of California Los Angeles: Sample 1; UCLA_2, University of California Los Angeles: Sample 2; UM_1, University of Michigan: Sample 1; UM_2, University of Michigan: Sample 2; USM, University of Utah School of Medicine; YALE, Yale Child Study Center.

Supplementary Table 2. Imaging protocols.

Sites	KKI	LEUVEN_1	MAX_MUN	NYU	OHSU	OLIN	PITT	SDSU
MRI Scanner	Philips Achieva	Philips	Siemens Magnetom Verio	Siemens Magnetom Allegra	Siemens Magnetom TrioTim	Siemens Magnetom Allegra	Siemens Magnetom Allegra	GE MR750
Magnetic field strength (T)	3	3	3	3	3	3	3	3
Field of view (mm)	256	230	192	240	240	220	200	220
Matrix	84×81	64×64	--	--	--	--	64×64	64×64
Number of slices	47	32	28	33	36	29	29	--
In-plane resolution (mm)	3.05×3.15	3.59×3.59	3.0×3.0	3.0×3.0	3.8×3.8	3.4×3.4	3.1×3.1	3.4×3.4
Slice thickness (mm)	3	4	4	4	3.8	4	4	3.4
Slice gap (mm)	0	0	--	--	--	--	0	0
TR (ms)	2500	1667	3000	2000	2500	1500	1500	2000
TE (ms)	30	33	30	15	30	27	25	30
Total scan time (mm:ss)	6:40	7:06	6:06	6:00	3:32	5:15	5:06	6:10
Flip angle	75	90	80	90	90	60	70	90
Slice acquisition order	Ascending	Ascending	--	--	--	--	Ascending	Ascending
Eyes during scan	Opened	Opened	Closed/Opened	Closed/Opened	Opened	Opened	Closed	Opened

Supplementary Table 2 continued.

	TRINITY	UCLA_1	UCLA_2	UM_1	UM_2	USM	YALE
MRI Scanner	Philips Achieva	Siemens Magnetom TrioTim	Siemens Magnetom TrioTim	GE Signa	GE Signa	Siemens Magnetom TrioTim	Siemens Magnetom TrioTim
Magnetic field strength (T)	3	3	3	3	3	3	3
Field of view (mm)	240	192	192	220	220	220	220
Matrix	80×80	64×64	64×64	--	--	--	--
Number of slices	38	34	34	40	40	40	34
In-plane resolution (mm)	3.0×3.0	3.0×3.0	3.0×3.0	3.4×3.4	3.4×3.4	3.4×3.4	3.4×3.4
Slice thickness (mm)	3.5	4	4	3	3	3	4
Slice gap (mm)	0.35	0	0	0	0	--	--
TR (ms)	2000	3000	3000	2000	2000	2000	2000
TE (ms)	28	28	28	30	30	28	25
Total scan time (mm:ss)	5:06	6:06	6:06	10:00	10:00	8:06	6:40
Flip angle	90	90	90	90	90	90	60
Slice acquisition order	Ascending	Ascending	Ascending	--	--	--	--
Eyes during scan	Closed	Opened	Opened	Opened	Opened	Opened	Opened

Abbreviations. KKI, Kennedy Krieger Institute; LEUVEN_1, University of Leuven: Sample 1; MAX_MUN, Ludwig Maximilians University Munich; NYU, NYU Langone Medical Center; OHSU, Oregon Health and Science University; OLIN, Olin, Institute of Living at Hartford Hospital; PITT, University of Pittsburgh School of Medicine; SDSU, San Diego State University; STANFORD, Stanford University; TRINITY, Trinity Centre for Health Sciences; UCLA_1, University of California Los Angeles: Sample 1; UCLA_2, University of California Los Angeles: Sample 2; UM_1, University of Michigan: Sample 1; UM_2, University of Michigan: Sample 2; USM, University of Utah School of Medicine; YALE, Yale Child Study Center.
