# Supplementary material for: Interaction between Functional Connectivity and Neural Excitability in Autism: A Novel Framework for Computational Modeling and Application to Biological Data
Source: Comput Psychiatr. 2023 Jan 20;7(1):14–29. doi: 10.5334/cpsy.93 (PMC11104370; doi:10.5334/cpsy.93)
Supplement: Supplementary File 3. — Supplementary Figure 1. [file cpsy-7-1-93-s3.pdf]

Supplementary Figure 1

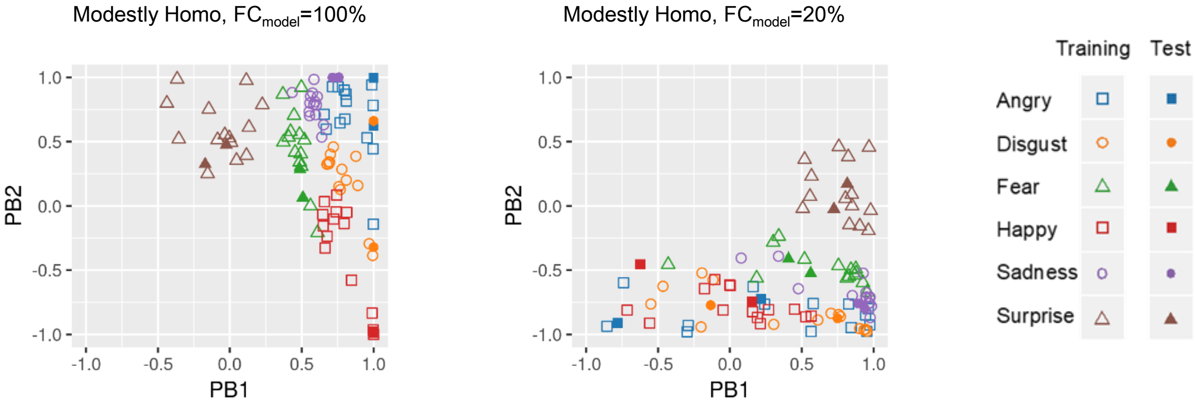

Note that FC_{model} =100% and FC_{model} =20% indicate that both higher-level FC_{model} and lower-level FC_{model} are 100% and 20%, respectively.
